# Acute Hypercalcemia Resulting From Antibiotic-Impregnated Calcium Sulfate Beads

**DOI:** 10.7759/cureus.100515

**Published:** 2025-12-31

**Authors:** Bryan Lopez, Vindeep Bhandari, Guilianne R Servano

**Affiliations:** 1 Department of Internal Medicine, David Geffen School of Medicine, University of California, Los Angeles, Los Angeles, USA

**Keywords:** calcium sulfate beads, hip arthroplasty, hypercalcemia, prosthetic joint infection, stimulan

## Abstract

An 86-year-old man with a history of chronic kidney disease sustained a hip fracture after a mechanical fall, which eventually led to a total hip arthroplasty (THA). During this THA, he was found to have a prosthetic joint infection and underwent debridement, antibiotics, and implant retention, where calcium sulfate beads (CSBs) were placed. He subsequently developed hypercalcemia, requiring intravenous fluids, diuretics, and calcitonin for treatment. This case highlights the development of hypercalcemia after the placement of CSBs, which is a known, although uncommon, side effect.

## Introduction

Calcium sulfate beads (CSBs) play a critical role in orthopedic surgery. The intraoperative use of these beads can serve as a bone void filler and provide local antibiotic delivery [1,2]. Once implanted, CSBs dissolve gradually over six to seven weeks, allowing for local prolonged antimicrobial protection and providing the scaffolding for proper bone healing [1]. This reduces the need for additional orthopedic interventions and also decreases the chances of adverse events such as osteomyelitis or joint infections [3]. This case report focuses on iatrogenic hypercalcemia, a relatively uncommon but recognized side effect of CSBs.

## Case presentation

An 86-year-old man with a history of stage 4 chronic kidney disease and benign prostatic hyperplasia sustained a right hip fracture following a mechanical fall. He underwent a right hip hemiarthroplasty for surgical correction. Several weeks later, he experienced another fall, which was complicated by the dislocation of the right hip. Orthopedic surgery then converted his hemiarthroplasty to a total hip arthroplasty (THA). Intraoperative cultures collected during the THA grew *Staphylococcus capitis* and *Staphylococcus epidermidis*. Due to this prosthetic joint infection (PJI), orthopedic surgery was performed, including debridement, antibiotics, and implant retention (DAIR) one week later. During this procedure, Stimulan CSBs (Biocomposites Ltd., Keele, UK) impregnated with 20 cc of vancomycin and tobramycin were placed. Additionally, a wound vacuum was applied to support wound healing. The patient tolerated the procedure well and remained hospitalized for just over a week before being transferred to a rehabilitation facility. Calcium level at date of discharge was 11.4 mg/dL.

Table 1 outlines the trend in renal function, specifically with serum creatinine and glomerular filtration rate (GFR), as well as calcium levels over the postoperative period. The placement of the CSBs occurred intraoperatively on postoperative day (POD) 0. On that day, the patient’s GFR and creatinine were at baseline, and his calcium levels were within normal limits. By POD 2, calcium levels began to rise, peaking at 11.4 mg/dL on POD 10, the day of hospital discharge. Although the calcium level was elevated, it was not critical, and the patient was deemed medically stable to transfer to a rehabilitation facility to continue physical therapy and complete a course of intravenous (IV) antibiotics as directed by the Infectious Disease consultants.

**Table 1:** Postoperative calcium levels

Postoperative day (placement of CSBs is day 0)	Estimated GFR, mL/minute	Creatinine, mg/dL (0.55-1.30 mg/dL)	Calcium, mg/dL (8.5-10.4 mg/dL)
0	25	2.46	8.7
1	23	2.59	8.7
2	21	2.86	9.7
3	21	2.87	9.9
4	21	2.84	10.4
5	20	2.98	10.7
6	20	2.97	9.9
7	20	3	10
8	19	3.04	11.1
9	20	3.01	10.4
10	19	3.02	11.4
11	19	3.12	11.5
14	15	3.67	13.8
15	17	3.41	12.1
18	20	2.94	9.9

Three days after arriving at the rehabilitation facility (POD 14), laboratory testing revealed a calcium level of 13.8 mg/dL, along with worsening creatinine and a decreased GFR. The patient was asymptomatic. He was treated with IV fluids, calcitonin, and subsequently IV diuretics. A diagnostic workup, including a renal ultrasound and a computed tomography scan of the abdomen and pelvis without contrast, was unremarkable for any acute abnormalities. Following these interventions, the patient’s calcium level normalized, and his renal function improved back to baseline. Given the clinical timeline and CSB placement, it was presumed that the patient was having iatrogenic hypercalcemia that can occur with these beads.

POD 0 is the day the CSBs were placed intraoperatively. POD 10 is the last day of the hospital stay. POD 11 is the first day at acute rehabilitation. POD 14 is the critically elevated calcium level and the day on which IV fluids, calcitonin, and furosemide were given to the patient. By POD 18, calcium levels have normalized.

## Discussion

This patient developed a PJI following the completion of a THA. PJI is a serious complication, occurring in 1%-2% of primary arthroplasties, and is associated with high morbidity and need for complex interdisciplinary treatment strategies [3]. Based on recommendations from the Infectious Disease and Orthopedic Surgery teams, he underwent a DAIR with placement of antibiotic-eluting CSBs. Often, this type of debridement leaves resected surfaces exposed to bacteria [1], with debris that may include small biofilm fragments, which can subsequently lead to further infection. To prevent this, local antibiotic delivery, in the form of CSBs, is preferred over systemic doses, which may be required to achieve high antimicrobial levels and can cause unacceptable side effects [1,2].

Historically, poly(methyl methacrylate) (PMMA) bone cement has been used for local antibiotic therapy in orthopedic surgery; however, the elution of antibiotics from PMMA is highly variable. Additionally, PMMA is nonbiodegradable and must be removed after infection management, as it could impair the healing of the debrided bone defect. CSBs have the advantage of being biodegradable, eliminating the need for a second surgery for removal [4].

In this case, Stimulan beads were selected as the CSB of choice. Stimulan is an absorbable calcium sulfate antibiotic carrier used to fill a bone void or defect of the skeletal system created by surgery, cysts, tumors, osteomyelitis, or traumatic injury [5]. It is absorbed in approximately 30-60 days. A study done by Kallala et al. involving the use of Stimulan beads in 755 patients noted the development of transient hypercalcemia in 5.4% of cases, with levels returning to normal five days postoperatively [6]. Similarly, a systematic review of 1,049 patients undergoing revision arthroplasty described the significant risk of transient hypercalcemia (4.2% of patients); however, it did note that cases of symptomatic hypercalcemia were infrequent [7].

According to the manufacturer, contraindications for Stimulan use include patients with renal impairment, hypercalcemia, severe vascular or neurological disease, uncontrolled diabetes, pregnancy, severe degenerative bone disease, and individuals who abuse alcohol or drugs [5]. Although renal impairment is stated as a complication, the manufacturer does not specify explicit renal thresholds, such as a GFR cutoff. Perhaps, establishing a standardized GFR cutoff should be recommended for dose adjustments, similar to most FDA-approved medications. This is important because the volume of CSBs used during surgery has been shown to potentially correlate with the subsequent severity of hypercalcemia [6,8]. Usually, 20-40 cc of Stimulan is used, with a mean volume of 25 cc [8]. The manufacturer of Stimulan recommends a dose of 20 cc; however, surgeons often exceed the manufacturer’s suggested volume, potentially increasing the risk of hypercalcemia, especially in patients who may have other risk factors as listed above.

## Conclusions

CSBs are commonly used in orthopedic surgery as both a bone void filler and as a way to provide local antibiotic therapy. In this case review, the patient developed hypercalcemia following CSB placement for a PJI after undergoing a THA. Clinicians should be aware of this potential complication. Monitoring serum calcium levels following CSB use should be considered to determine if this side effect occurs, particularly in patients with underlying risk factors for hypercalcemia. For patients with compromised renal function, dose adjustment should be considered, and perhaps a standardized GFR cutoff could be recommended.
